# Examination of Trends in Diabetes Incidence Among Children During the COVID-19 Pandemic in Ontario, Canada, From March 2020 to September 2021

**DOI:** 10.1001/jamanetworkopen.2022.23394

**Published:** 2022-07-25

**Authors:** Rayzel Shulman, Eyal Cohen, Therese A. Stukel, Christina Diong, Astrid Guttmann

**Affiliations:** 1 Division of Endocrinology, Hospital for Sick Children, Toronto, Ontario, Canada; 2 Division of Paediatric Medicine, Hospital for Sick Children, Toronto, Ontario, Canada; 3 ICES, Toronto, Ontario, Canada

## Abstract

This cross-sectional study uses health administrative data to examine trends in diabetes incidence among children during the COVID-19 pandemic in Ontario, Canada.

## Introduction

A recent study reported an association between COVID-19 infection and new-onset diabetes among people younger than 18 years in the US.^1^ The resulting media coverage was extensive, although some experts have criticized the study methods and conclusion validity. There is no clear mechanism by which COVID-19 infection might cause new-onset diabetes.^2^ Kamrath et al^3^ recently reported an increase in type 1 diabetes incidence among children in Germany during the pandemic. They did not observe an association of COVID-19 and increased type 1 diabetes incidence in the months after infection or an increase in the frequency of autoantibody-negative type 1 diabetes, which prompts the question of whether COVID-19 infection is associated with incident type 1 diabetes.^3^ Given the challenges of ascertaining a COVID-19 infection history for children with new-onset diabetes, additional population-based studies investigating changes in diabetes incidence among children during the pandemic are needed. Canada has one of the highest incident rates of type 1 diabetes worldwide. Therefore, this study examined whether diabetes incidence increased during the COVID-19 pandemic among children and youths (aged <18 years) in Ontario, Canada.

## Methods

For this population-based, repeated cross-sectional study, data use was authorized under Section 45 of Ontario’s Personal Health Information Protection Act and therefore did not require research ethics board review or informed consent. The study followed the STROBE reporting guideline.

We used health administrative data (January 2017 to September 2021) linked using unique encoded identifiers, held and analyzed at ICES (formerly the Institute for Clinical Evaluative Sciences) in Ontario, Canada. We included all children and youth (aged 1-17 years) eligible for universal health care insurance (all legal residents) on January 1 of each year (2017 to -2021).

Ontario has a population of approximately 14.8 million people; 3 million are younger than 18 years. Between November 2020 and April 2021, an estimated 3.3% of children in Ontario had SARS-CoV-2 infection.^4^ We used generalized estimating equations for Poisson regression to model 3-year pre–COVID-19 rates adjusting for age group, sex, pre–COVID-19 month, and secular trend. We then used these models to estimate expected post–COVID-19 monthly rates (95% CIs) using 2-sided hypothesis tests (eMethods in the Supplement).^5^ The exposure was the pandemic era starting in March 2020, and the outcome was new diabetes diagnosis. Statistical analyses were conducted using SAS version 9.4 (SAS Institute).

## Results

**Figure 1.**
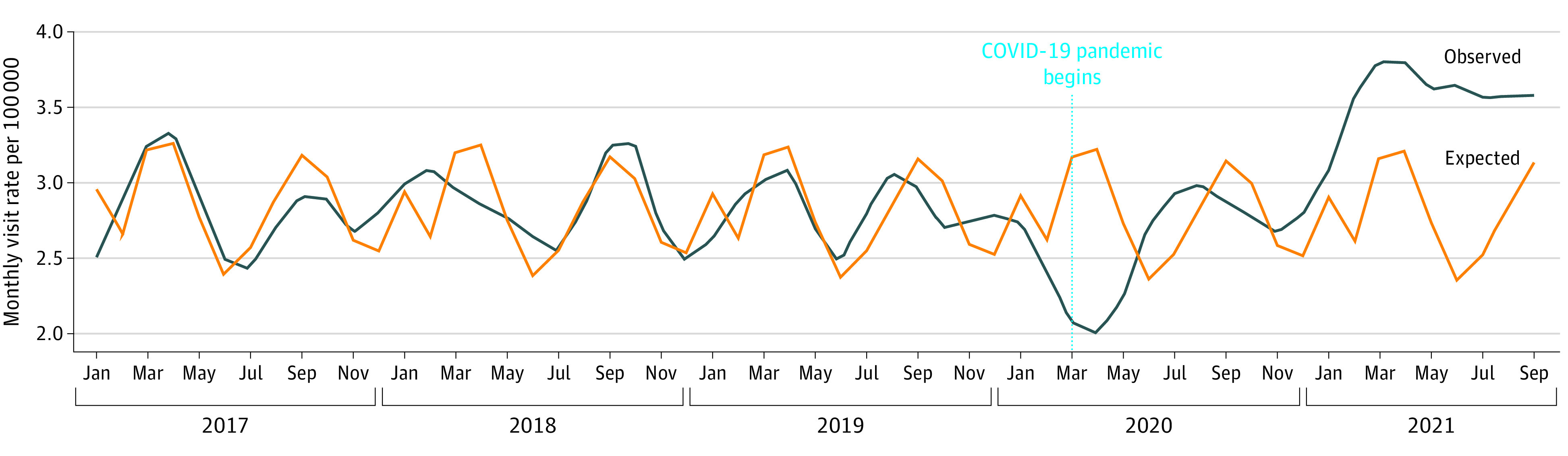
Observed vs Expected Incidence of Diabetes Among Children and Adolescents (Aged 1-17 Years) in Ontario, Canada, From January 2017 to September 2021, by Month

**Figure 2.**
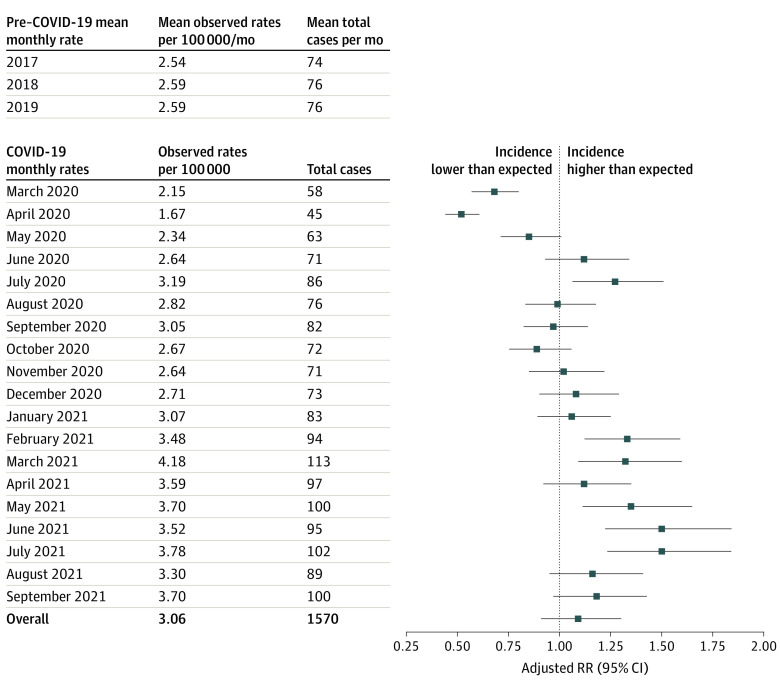
Mean New Diabetes Diagnoses Among Children and Adolescents in Ontario, Canada, Before and During the COVID-19 Pandemic and Adjusted Relative Rates of Incident Diabetes During the Pandemic, by Month Mean monthly new diabetes diagnoses are presented for each year before the pandemic and for each month during the pandemic (2017-2021). RR denotes relative rate.

There were 2 700 178 children in the 2021 cohort; the mean (SD) age was 9.2 (4.9) years, and 48.7% were girls. Overall, there was no difference in observed vs expected relative rates (RRs) of new diabetes presentations (RR, 1.09 [95% CI, 0.91-1.30]) (Figure 1). However, RRs of new diabetes presentations decreased in the first 3 months of the pandemic (15%-32% lower in March to May 2020), with a subsequent increase to higher-than-expected rates (33%-50% higher in February to July 2021; Figure 2).

## Discussion

In this cross-sectional study, we observed a slightly higher but nonsignificant increase in diabetes incidence among children during the COVID-19 pandemic. Our overall rate ratio is similar to that of Kamrath et al,^3^ who reported a 1.15-fold increase in type 1 diabetes incidence among children in Germany during the pandemic.^3^ Limitations of our study include its smaller population and therefore lower power; thus, we cannot rule out a 1.3-fold increase in RRs. An advantage of our study is that we report monthly variations in post–COVID-19 diabetes incidence showing a decline then an increase in rates, suggesting possible delays in diabetes diagnosis for children early in the pandemic with a catch-up effect. Although we are unable to differentiate type 1 and 2 diabetes, 95% of children with diabetes in Ontario have type 1.^6^ The lack of both an observable increase in overall diabetes incidence among children during the 18-month pandemic restrictions and a plausible biological mechanism calls into question an association between COVID-19 and new-onset diabetes. Given the variability in monthly RRs, additional population-based, longer-term data are needed to examine the direct and indirect effects of COVID-19 and diabetes risk among children.

## Notes

Supplement. **eMethods.** Complete Methods
eReferences
