# Supplementary material for: Examination of Trends in Diabetes Incidence Among Children During the COVID-19 Pandemic in Ontario, Canada, From March 2020 to September 2021
Source: JAMA Netw Open. 2022 Jul 25;5(7):e2223394. doi: 10.1001/jamanetworkopen.2022.23394 (PMC9315418; doi:10.1001/jamanetworkopen.2022.23394)
Supplement: Supplement. — eMethods. Complete Methods eReferences [file jamanetwopen-e2223394-s001.pdf]

Supplementary Online Content

Shulman R, Cohen E, Stukel TA, Diong C, Guttmann A. Examination of trends in diabetes incidence among children during the COVID-19 pandemic in Ontario, Canada, from March 2020 to September 2021. *JAMA Netw Open*. 2022;5(7):e2223394. doi:10.1001/jamanetworkopen.2022.23394

eMethods. Complete Methods

eReferences

This supplementary material has been provided by the authors to give readers additional information about their work.

© 2022 Shulman R et al. *JAMA Network Open*.

eMethods. Complete Methods

Data Sources

We used the following administrative datasets at ICES (formerly the Institute of Clinical Evaluative Sciences) in Toronto, Canada: the Hospital Discharge Abstract Database (information on discharges from acute care facilities); the Ontario Health Insurance Plan Database (physician billing claims); the National Ambulatory Care Reporting System (information on emergency department visits); the Registered Persons Database (demographics and vital statistics including outmigration of all legal residents in Ontario); and the Ontario Diabetes Database (ODD), a population-based diabetes registry derived using administrative data, and validated in children (83% sensitivity, 99% specificity)(1).

Coding Definitions for Diabetes

We ascertained individuals with a new diabetes diagnosis if they had a diabetes code ICD-10: E10-E14 in the Canadian Institute for Health Information-Discharge Abstract Database (hospitalizations) or National Ambulatory Care Reporting System (emergency department visits). To be counted as a new diabetes diagnosis, the individual also must have no record in the Ontario Diabetes Dataset and no Ontario Health Insurance Plan (physician outpatient billings) diagnosis code 250 or any insulin/diabetes management fee codes in the previous 2 years, excluding the 1 month prior. The insulin/diabetes management fee codes are as follows: K029, G500, G514, G520, K030, K045, K046, and Q040. The validated algorithm for inclusion in the Ontario Diabetes Dataset is ≥4 Ontario Health Insurance Plan with diagnosis code 250 in a two-year period OR ≥1 Ontario Health Insurance Plan (insulin/diabetes management fee codes) (Q040/K029/K030)(1). Since this algorithm requires 2 years of observation for ascertainment, we cannot use this registry for real time monitoring of incidence.

For the current study, we used the method described above using hospitalization and ED visits only to ascertain incident diabetes in a shorter time frame. We validated that there were no differences in the proportion of new diabetes diagnoses identified using the two methods across all pre-pandemic years (2017-2019) and a majority of children with a new diagnosis have an ED visit or admission for diabetes diagnosis and management.

Model Description

We used similar methods as in previous work to compute rates and to model post-COVID-19 expected rates, with some minor modifications (2). Briefly, we used Poisson generalized estimating equations (GEE) models for clustered count data to model 3-year pre-COVID-19 monthly trends and used these to predict expected post-COVID-19 trends in the absence of restrictions. The unit of analysis was the age group-sex-month stratum. The dependent variable was the stratum-specific count of events to the population in the stratum; the offset was the log of the stratum-specific population. The pre-COVID-19 model included age group-sex indicators, pre-COVID-19 month indicators to model seasonal variations with April as the reference month, and a continuous linear term of months since January 1, 2017 to estimate the general trend in diabetes incidence through March 1, 2020.

We computed the expected post-COVID-19 diabetes incidence (and 95% confidence intervals [CI]) by applying the linear combination of pre-COVID-19 regression coefficients to the post-COVID-19 age-sex-month strata and exponentiating. The adjusted relative change in post-COVID-19 diabetes incidence was expressed as the ratio of observed to expected rates and calculated by exponentiating the difference of observed and expected post-COVID-19 log rates and corresponding 95% confidence intervals.

Our study followed the Strengthening the Reporting of Observational Studies in Epidemiology (STROBE) reporting guideline.

ICES is a prescribed entity under Ontario’s Personal Health Information Protection Act (PHIPA). Section 45 of PHIPA authorizes ICES to collect personal health information, without consent, for the purpose of health planning, monitoring, and evaluation. Projects that use data collected by ICES under section 45 of PHIPA, and use no other data, are exempt from REB review. The use of the data in this project is authorized under section 45 and approved by ICES’ Privacy and Legal Office.

© 2022 Shulman R et al. *JAMA Network Open*.

eReferences

1. Guttmann A, Nakhla M, Henderson M, To T, Daneman D, Cauch-Dudek K, et al. Validation of a health administrative data algorithm for assessing the epidemiology of diabetes in Canadian children. Pediatr Diabetes. 2010;11(2):122-8.

2. Saunders N, Guttmann A, Brownell M, Cohen E, Fu L, Guan J, et al. Pediatric primary care in Ontario and Manitoba after the onset of the COVID-19 pandemic: a population-based study. CMAJ open. 2021;9(4):E1149-e58.

© 2022 Shulman R et al. *JAMA Network Open*.
